# A narrative review of research status and prospects of mindfulness-based interventions in schizophrenia

**DOI:** 10.3389/fpsyg.2026.1930111

**Published:** 2026-10-12

**Authors:** Jingli Xin, Jianli Zhu, Huipeng Lu

**Affiliations:** The Third People’s Hospital of Zhongshan, Zhongshan, China

**Keywords:** emotion regulation, insight, mindfulness-based interventions, schizophrenia, stigmatization

## Abstract

Schizophrenia patients often experience frailty due to persistent hallucinations, delusions, negative symptoms, and social stigmatization, severely impacting their quality of life and psychosocial functioning. Recently, mindfulness intervention therapy has emerged as a supplementary treatment for residual symptoms and functional decline in schizophrenia. Numerous controlled studies, systematic reviews, and meta-analyses have examined the effects of mindfulness-based interventions on schizophrenia. Despite this, the mechanisms through which mindfulness affects schizophrenia remain unclear. This review incorporates qualitative research and correlation analyses to explore the relationships among mindfulness traits, psychological and neurological changes, symptom improvement, and functional enhancement, identifying unresolved scientific questions. Additionally, the article examines research findings on safety, digitalization, and mobile health. Findings indicate a complex relationship between mindfulness and patients’ insight, self-compassion, and stigmatization. Enhancing attention control and emotional regulation may underlie the effectiveness of mindfulness interventions for schizophrenia. Increased insular activity and reduced default mode network connectivity might contribute to mindfulness’s potential benefits for these patients. This study offers a foundation for future mindfulness intervention programs for schizophrenia and suggests directions for further research.

## 1 Introduction

Schizophrenia is a severe mental illness. While drug treatments effectively address positive symptoms, they offer limited relief for refractory hallucinations and delusions and are even less effective for negative symptoms (Cella et al., 2023; Raffard and Bortolon, 2021). Persistent social stigma, along with ongoing hallucinations, delusions, and negative symptoms, debilitates patients and significantly impairs their quality of life and psychosocial functioning (Hochheiser et al., 2020).

In recent years, the need for improved strategies to address residual symptoms and functional decline in schizophrenia has become evident. Mindfulness-based therapy (MBT), a “third-wave” psychotherapy, has emerged as a promising adjunctive treatment. It has gained significant attention for its effectiveness in enhancing symptoms and psychosocial functioning in schizophrenia patients through conscious, non-judgmental awareness of the present moment (Reangsing et al., 2024; Plencler et al., 2025b).

Previous review articles primarily conducted meta-analyses on the effects of mindfulness-based interventions (MBI) for schizophrenia patients, focusing solely on effectiveness and effect size. The mechanism through which these interventions impact schizophrenia remains unclear. This review includes qualitative studies and correlation analyses to address this gap. It comprehensively examines the current research on MBI for schizophrenia, offering a reference for developing intervention programs and guiding future research directions.

## 2 Search strategy

On April 12, 2026, we searched PubMed, Web of Science, APA PsycINFO, and Scopus for literature from 2021 to 2026 using the terms “mindfulness,” “mindfulness-based stress reduction,” “mindfulness-based cognitive therapy,” “mindfulness-based interventions,” “schizophrenia,” “schizoaffective disorder,” and “psychosis.” Zotero was used for literature management. Two reviewers manually assessed the reference list, removed duplicates, and evaluated studies based on inclusion and exclusion criteria. Exclusion criteria were: (1) not in English; (2) not in peer-reviewed journals; (3) focused solely on long-term meditation practice; (4) considering mindfulness only as a characteristic; (5) treatment methods not primarily mindfulness-focused, such as dialectical behavior therapy and acceptance and commitment therapy; (6) subjects with mental disorders other than schizophrenia; (7) targeting non-adult or elderly populations.

## 3 Results

### 3.1 Search results

According to the retrieval criteria, 1,039 articles were obtained from various databases (PubMed = 147, Web of Science = 317, APA PsycINFO = 202, Scopus = 373). After removing duplicates, non-peer-reviewed articles, and non-English papers, 351 studies remained. These studies were then screened based on their titles and abstracts to meet the inclusion and exclusion criteria. Ultimately, 39 articles were included in the research summary. These studies have demonstrated the use of MBI in patients with schizophrenia, highlighting associated mental, psychological, and physiological changes, they have also investigated the underlying mechanisms and relationships involved.

### 3.2 Intervention methods

The primary mindfulness-based intervention methods include mindfulness-based stress reduction (MBSR), mindfulness-based cognitive therapy (MBCT), mindfulness-based psychological program (MBPP), mindfulness meditation, and mindfulness-based yoga therapy.

### 3.3 Clinical efficacy

#### 3.3.1 Positive symptoms

Recent studies have produced conflicting results on the efficacy of MBI for positive symptoms in schizophrenia. Liu et al. (2021) and Su et al. (2024) found that these interventions significantly reduced positive symptoms in such patients. Wang et al. (2021) reported that mindfulness meditation notably decreased hallucination and delusion symptoms compared to conventional treatments in chronic schizophrenia patients. Xue et al. (2024) observed that an 8-month daily intensive mindfulness meditation intervention was more effective in reducing hallucinations and delusions in male patients with refractory symptoms than a 3-month intervention. Hahne et al. (2026) demonstrated that a mindful present-focused yoga group intervention had a medium-to-large effect on positive symptoms and general psychopathology in schizophrenia patients. However, reviews by Fattahi et al. (2021) and Plencler et al. (2025b) indicated that the effect on positive symptoms was relatively small. Thus, further research is necessary to confirm the impact of MBI on positive symptoms in schizophrenia.

#### 3.3.2 Negative symptoms

Recent research consistently indicates that MBI significantly alleviate negative symptoms in patients with schizophrenia. A four-week mindfulness-based group intervention by Böge et al. (2021) and a mindful present-focused yoga group intervention by Hahne et al. (2026) both demonstrated medium to large reductions in negative and depressive symptoms before and after the interventions. Additionally, four systematic reviews (Chai et al., 2022; Liu et al., 2021; Plencler et al., 2025b; Sabé et al., 2024) confirmed the significant reduction of negative symptoms through MBI. Liu et al. (2021) found that the improvements in negative symptoms persisted for 3 to 6 months, alongside reductions in depressive symptoms. Chai et al. (2022) reported a small to large effect on negative symptoms improvement (SMD = −0.59, 95% CI: −0.87 to −0.32). Overall, MBI reliably improve negative symptoms in schizophrenia patients. The systematic reviews conducted by Plencler et al. (2025b) and Sabé et al. (2024) both incorporated the randomized controlled trial studies by Böge et al. (2021) in their analyses. Consequently, the strength of the evidence may be overstated. Caution is advised in the interpretation of these findings.

#### 3.3.3 Insight, self-compassion, and stigmatization

Mindfulness has a complex, bidirectional relationship with insight, self-compassion, and stigmatization in schizophrenia patients. Two systematic reviews have examined the impact of MBI on these patients’ insight. A meta-analysis of 24 studies involving 1,632 participants found that MBIs significantly improved insight (*g* = 1.21, 95% CI: 0.88 to 1.55), particularly when homework was included (Reangsing et al., 2024). Another review of 13 studies with 2,899 participants also reported that the MBI group had significantly better insight than the control group (SMD = 1.05, 95% CI: 0.67 to 1.43, *p* < 0.00001) (Gao et al., 2025). Enhanced insight positively influences the reduction of aggression, hostility, and the effects of mind-wandering on mental distress in schizophrenia patients (Mervis et al., 2022; Rodolico et al., 2023).

Stigmatization significantly impedes the recovery of schizophrenia patients. A meta-analysis by Gao et al. (2025) found that MBI effectively reduce stigmatization in these patients. Karahan Agu and Orak (2025) implemented self-compassion-oriented mindfulness psychoeducation for schizophrenia patients and assessed them using the Internalized Stigma of Mental Illness (ISMI) scale. Their findings revealed that the intervention group experienced a significant reduction in total ISMI scores and sub-dimensions such as alienation, stereotype endorsement, perceived discrimination, and social withdrawal compared to the control group, with a 17.3% decrease in the total ISMI score (ηp^2^ = 0.173, *p* = 0.006). Increased self-compassion can mitigate over-identification in schizophrenia patients, thus reducing stigmatization (Toshi et al., 2025). After two 8-week sessions of MBCT for female schizophrenia patients, the intervention group showed a significant decrease in stigmatization, evidenced by changes in stigma perception and reduced withdrawal and avoidance behaviors (Tang et al., 2021; Xue et al., 2025). The meta-analysis conducted by Gao et al. (2025) incorporated the randomized controlled trial by Tang et al. (2021) for its analysis. Consequently, the strength of the evidence may be overstated. Caution is advised in interpretation.

Enhanced insight in schizophrenia patients can reduce self-compassion (Hochheiser et al., 2020) and increase stigmatization (Zhang S. et al., 2025). Thus, further research is essential to explore how MBI affect insight, self-compassion, and stigmatization in these patients. Fortunately, higher levels of trait mindfulness and perceived social support significantly mitigate the negative impact of increased insight on stigmatization (Zhang S. et al., 2025). Therefore, mindfulness interventions should prioritize enhancing trait mindfulness over state mindfulness. Developing tailored mindfulness programs for schizophrenia patients is crucial to improve treatment and rehabilitation outcomes.

#### 3.3.4 Attention and emotional regulation

Mindfulness may enhance attention regulation, potentially improving symptoms in schizophrenia patients. In a study, participants with schizophrenia were divided into four groups: mindfulness, attention-concentration, attention-avoidance, and a control group. They completed an ambiguous auditory task, and researchers evaluated distress levels and the content of heard words. Results showed that the mindfulness group experienced less distress than the attention-concentration and control groups. Additionally, the attention-avoidance and attention-concentration groups heard fewer words than the control group. This suggests that targeting attention could be effective in treating auditory hallucinations in psychosis. Moreover, manipulating attention may influence the frequency of auditory hallucinations and related distress (Madani et al., 2023). A structural equation model by Tagami and Imaizumi (2023) indicates that mindfulness characteristics—observing, acting with awareness, and non-judging—mediate the relationship between schizotypy and hallucinatory experiences (CFI = 0.964, SRMR = 0.044, GFI = 0.974). Attention to current experiences may be crucial for understanding the cognitive mechanisms behind schizotypal and schizophrenia-related hallucinations. Recently, Wiessner et al. (2025) conducted an 8-week mindfulness-based health promotion course with healthy participants. They found that mindfulness improved wellbeing and stress management by regulating pre-attentional and attentional processes, reducing abnormal salience, and promoting decentering. However, further research is needed to understand how mindfulness enhances attention control in schizophrenia patients.

A review and qualitative study have highlighted the effects of MBI on emotion regulation in schizophrenia patients. Plencler et al. (2025b) analyzed 11 studies and found that group mindfulness interventions positively influenced emotion regulation in these patients. Similarly, Lam et al. (2021) conducted a qualitative analysis on a MBPP involving 24 patients. Participants reported learning effective emotion regulation methods through the MBPP. Depression mediates the link between emotion regulation and self-stigma in schizophrenia patients, with a significant mediation effect (*B* = 0.095, *β* = 0.458, *p* = 0.001, 95%CI: 0.04 to 0.14). Enhancing emotion regulation skills can reduce both depression and self-stigma in these patients (Rayan, 2025; Raz et al., 2025). Additionally, impairments in emotion recognition and regulation are linked to both positive and negative symptoms of schizophrenia (Lee and Yu, 2023; Raugh and Strauss, 2022). Anxiety disorders also contribute to the development of delusions in schizophrenia patients (Mackintosh et al., 2021).

#### 3.3.5 Hope level, psychological wellbeing, function, and readmission rate

Two intervention experiments examined the effects of MBSR training on the hope levels of patients with schizophrenia. Compared to the psychoeducation and control groups, this training proved more effective in enhancing hope levels (Özdemir and Kavak Budak, 2022; Zheng and Zhi, 2022). Additionally, these experiments reported significant improvements in the psychological wellbeing and functional levels of these patients. Furthermore, two systematic reviews, encompassing 33 randomized controlled studies, confirmed that MBI significantly enhanced the psychological wellbeing of schizophrenia patients (Plencler et al., 2025b; Sabé et al., 2024). The systematic review conducted by Sabé et al. (2024) incorporated the study by Özdemir and Kavak Budak (2022) for analysis, potentially leading to an overestimation of the strength of the evidence. Therefore, caution is advised in interpreting the findings. Sabé’s findings also indicated a reduction in the readmission rate for these patients. A meta-analysis of nine controlled experiments by Liu et al. (2021) supported Sabé’s results, showing that MBI decreased the readmission time for schizophrenia patients (Sabé et al., 2024).

Recent studies have assessed the effectiveness of MBI in enhancing social, overall, and occupational functions, as well as disease management in patients with schizophrenia. Plencler et al. (2025b) conducted a systematic review and found that mindfulness-based group interventions positively influenced the social and occupational functions of these patients. Böge et al. (2021) carried out a randomized controlled trial with 40 participants, evaluating a four-week mindfulness group therapy for schizophrenia patients. Their findings indicated a medium-to-large improvement in social function and quality of life for those receiving mindfulness therapy combined with regular treatment, compared to a medium-to-small improvement in the regular treatment group. Reangsing et al. (2024) conducted a systematic review and meta-analysis of 24 studies involving 1,632 participants. They found that the overall function of the mindfulness-based intervention group significantly improved compared to the control group (*g* = 1.28, 95% CI: 0.50 to 2.05), suggesting a medium-to-large effect. Additionally, Lam et al. (2021) reported through qualitative analysis that a MBPP enhanced disease management abilities in schizophrenia participants. Böge et al. (2021) and Lam et al. (2021) both analyzed the same study cohort, while the systematic review by Plencler et al. (2025b) incorporated the findings from Böge et al. (2021). Caution is advised in interpretation.

#### 3.3.6 Changes in cranial nerves

In recent years, electroencephalography and functional imaging have been employed to investigate neural changes in schizophrenia patients following MBI and meditation. Fattahi et al. (2021) found that patients undergoing MBT showed increased volume and activity in the insula and anterior cingulate cortex, suggesting potential benefits of MBT for these patients. Kong et al. (2025) conducted an 8-month meditation intervention with 30 chronic schizophrenia patients. At 3 and 8 months post-intervention, participants exhibited significantly enhanced functional connectivity between the left dorsolateral anterior insula, left posterior insula, and right orbital part of the inferior frontal gyrus. Additionally, PANSS scale scores decreased significantly, indicating overall improvement in psychiatric symptoms. This study highlighted the critical role of mindfulness meditation in boosting insular activity to alleviate symptoms in schizophrenia patients (Fox et al., 2016).

One experiments utilized mindfulness-based real-time functional magnetic resonance imaging neurofeedback (MBNF) to explore cerebral nerve changes in schizophrenia patients. Zhang J. et al. (2025) discovered that a single MBNF session could decrease DMN connectivity and alleviate auditory hallucinations related to baseline DMN connectivity. These findings suggest that mindfulness interventions reducing DMN hyperconnectivity may help diminish auditory hallucinations in schizophrenia patients.

### 3.4 Online and virtual reality mindfulness training

With advancements in digital technology, research has increasingly focused on online and virtual reality mindfulness training for schizophrenia patients, enhancing their rehabilitation process. Andrade et al. (2025) conducted a systematic review of an online user-led psychiatric auxiliary treatment system, finding that online mindfulness intervention effectively improves schizophrenia symptoms. In a separate study, McDonald et al. (2024) performed a 40-day randomized controlled trial of mindfulness-based online intervention (MBI) on patients with high suspicion/paranoia schizophrenia. The evaluation revealed no impact of MBI on trait paranoia, but a reduction in VR-induced state paranoia was observed, with a medium to large effect size (63). Overall, online mindfulness interventions have proven effective for symptom improvement in schizophrenia patients. Additionally, three studies on mindfulness-based virtual reality training interventions demonstrated significant improvements in psychiatric symptoms, stress, anxiety, depression, and cognitive function in schizophrenia patients (Cechnicki et al., 2025; Plencler et al., 2024, 2025a).

### 3.5 Safety

In recent years, limited studies have explored the safety of MBI for schizophrenia patients. Lam et al. (2021) conducted semi-structured interviews with 8 schizophrenia patients one week after completing MBPP. Five participants reported negative experiences, including irritability, depression, and fear, while three had positive experiences. Plencler et al. (2025a) implemented a virtual reality mindfulness training for schizophrenia patients, finding that 8% (2 cases) experienced anxiety and 4% (1 case) reported increased stress. Conversely, another study by Plencler et al. (2025b) found mindfulness-based group interventions safe for schizophrenia patients, with no serious adverse effects reported. Overall, MBI have been deemed safe for schizophrenia patients.

## 4 Summary and outlook

MBI positively impact negative symptoms, anxiety, depression, stress, and cranial nerve function in schizophrenia patients. They enhance insight, hope, psychological wellbeing, and psychosocial function while reducing stigmatization and readmission rates. However, their effects on positive symptoms remain debated. Online and virtual reality mindfulness training also show significant benefits. Attention control and emotion regulation likely serve as the underlying mechanisms for these interventions. Although some studies report negative effects, no serious adverse consequences have been found, confirming the overall safety of mindfulness training.

As mentioned above, anxiety is a causal factor in the development of delusions, and abnormal insight is a key process in the emergence of delusions in individuals with schizophrenia (Tulver et al., 2023). Mindfulness may reduce delusional symptoms in individuals with schizophrenia by enhancing emotional regulation—through increased insight and enhanced insular activity—thereby alleviating anxiety. At the same time, mindfulness may reduce hyperconnectivity in the default mode network (DMN) of individuals with schizophrenia by enhancing attentional control, thereby reducing hallucinatory symptoms. On the other hand, mindfulness is positively correlated with intrinsic motivation (Li et al., 2023); higher levels of mindfulness are associated with stronger intrinsic motivation, which may explain why mindfulness is effective in improving negative symptoms in individuals with schizophrenia. Higher levels of trait mindfulness can significantly reduce the negative impact of increased insight on the stigmatization of individuals with schizophrenia; therefore, clinically, it is important not only to provide training in state mindfulness but also to focus on enhancing the level of trait mindfulness in individuals with schizophrenia.

Moreover, this article presents some limitations. Initially, the literature reviewed here is confined to the period from 2021 to 2026, potentially leading to the exclusion of pivotal early studies and reviews in the field. Additionally, the mindfulness-based intervention strategies encompassed in this study consist of mindfulness-based stress reduction therapy, mindfulness-based cognitive therapy, mindfulness-based mental health education, mindfulness-based meditation, and mindfulness-based yoga therapy, exhibiting notable diversity among these approaches. Lastly, there are constraints related to the limited number of research alternatives and variations in research quality.
